# The Biological and Clinical Significance of Glutaminase in Luminal Breast Cancer

**DOI:** 10.3390/cancers13163963

**Published:** 2021-08-06

**Authors:** Brendah K. Masisi, Rokaya El Ansari, Lutfi Alfarsi, Madeleine L. Craze, Natasha Jewa, Andrew Oldfield, Hayley Cheung, Michael Toss, Emad A. Rakha, Andrew R. Green

**Affiliations:** 1 Nottingham Breast Cancer Research Centre, Academic Unit for Translational Medical Sciences, School of Medicine, University of Nottingham Biodiscovery Institute, University Park, Nottingham NG7 2RD, UK; msxbkma@nottingham.ac.uk (B.K.M.); rokayaelansari@gmail.com (R.E.A.); alfarsil@yahoo.com (L.A.); mszmc@nottingham.ac.uk (M.L.C.); mzytnj@nottingham.ac.uk (N.J.); andrewoldfield1998@gmail.com (A.O.); mzyhcc@nottingham.ac.uk (H.C.); michael.toss@nottingham.ac.uk (M.T.); emad.rakha@nottingham.ac.uk (E.A.R.); 2 Cellular Pathology, Nottingham University Hospitals NHS Trust, Hucknall Road, Nottingham NG5 1PB, UK

**Keywords:** glutaminase, DCIS, IBC, prognosis

## Abstract

**Simple Summary:**

Certain nutrients are needed by cancers to grow. Some breast cancers need the nutrient glutamine to grow and without it they don’t grow as quickly. In this study, we wanted to know the role of an enzyme, glutaminase, which is a substance produced by the body that breaks down glutamine so it can be used by cancers to grow. This enzyme occurs as two different types but we don’t know what their roles are in breast cancer. We therefore looked at the two types of enzyme in over 7000 breast cancers. We found that patients with high amounts of enzyme in early forms of breast cancer died earlier. Therefore, this enzyme has an important role in breast cancer and could be used to identify cancers which will get worse. We also think that using a drug to stop this enzyme will stop cancers growing. More studies are needed to confirm this.

**Abstract:**

The glutamine metabolism has a key role in the regulation of uncontrolled tumour growth. This study aimed to evaluate the expression and prognostic significance of glutaminase in luminal breast cancer (BC). The glutaminase isoforms (GLS/GLS2) were assessed at genomic/transcriptomic levels, using METABRIC (*n* = 1398) and GeneMiner datasets (*n* = 4712), and protein using immunohistochemistry in well-characterised cohorts of Oestrogen receptor-positive/HER2-negative BC patients: ductal carcinoma in situ (DCIS; *n* = 206) and invasive breast cancer (IBC; *n* = 717). Glutaminase expression was associated with clinicopathological features, patient outcome and glutamine-metabolism-related genes. In DCIS, GLS alone and GLS+/GLS2- expression were risk factors for shorter local recurrence-free interval (*p* < 0.0001 and *p* = 0.001, respectively) and remained prognostic factors independent of tumour size, grade and comedo necrosis (*p* = 0.0008 and *p* = 0.003, respectively). In IBC, *GLS* gene copy number gain with high mRNA expression was associated with poor patient outcome (*p* = 0.011), whereas high GLS2 protein was predictive of a longer disease-free survival (*p* = 0.006). Glutaminase plays a role in the biological function of luminal BC, particularly GLS in the early non-invasive stage, which could be used as a potential biomarker to predict disease progression and a target for inhibition. Further validation is required to confirm these observations, and functional assessments are needed to explore their specific roles.

## 1. Introduction

Metabolic reprogramming has been recognised as a hallmark of cancer [1]. Malignant transformation and progression require alteration of signalling pathways related to cellular metabolism to meet the demand for both energy and biomass for proliferating malignant cells. Glutamine is the second most utilised source of energy after glucose used by cancer cells to support tumour cell proliferation and survival, and some breast cancers are known to exhibit glutamine addiction [2]. Glutamine plays a role in the replenishment of biosynthetic intermediates to maintain a functioning tricarboxylic-acid (TCA) cycle, and it allows for the synthesis of macromolecules and antioxidants for rapidly proliferating cells [3,4]. Glutamine is catabolised to glutamate by the mitochondrial enzyme glutaminase, which presents as two isoforms; kidney-type (GLS/KGA) and liver-type (GLS2/LGA) [5].

Both the prognostic and the therapeutic significance of these two glutaminase isoforms remain an active area of research. GLS is the main isoform expressed in cancer cells, and there is increasing evidence suggesting it plays an important role in carcinogenesis and tumour progression in various solid cancers. Previous studies have established that high GLS correlates with higher rates of tumour growth and is associated with advanced tumour stage and poor patient outcomes [6,7,8]. In contrast, although studies are limited on GLS2, it tends to have opposing functions, as it is markedly increased in tumours that are more differentiated and less aggressive [9,10]. High GLS2 expression is associated with a significantly longer survival time in hepatocellular carcinoma [11]. 

In breast cancer (BC), GLS is expressed at different levels in molecular subtypes and appears to play an important role in the aggressive subclass of luminal BC in addition to triple-negative BC (TNBC) [12]. It has also been observed that patients with high *GLS* but not *GLS2* mRNA expression in highly proliferative luminal BC have the worst patient outcome compared with those classified as low proliferative [13]. BC is a heterogeneous group of diseases with histological types and metabolic pathways together sustaining the initiation and progression [14,15]. The progression from ductal carcinoma in situ (DCIS) to invasive disease is a complex multifactorial process that involves different mechanisms, including metabolic pathways. Whilst glutamine dependency has been confirmed in TNBC, there remains a need to explore the role of GLS isoforms in luminal Oestrogen receptor-positive (ER + ) and human epidermal growth factor receptor 2-negative (HER2-) breast tumours which show higher glutamine metabolic activity in in vitro studies [16]. The prognostic significance of GLS isoforms in luminal DCIS and invasive disease also remain to be validated. Therefore, we hypothesised that both GLS and GLS2 play a role in the tumour progression and prognosis in luminal BC. This study aimed to assess the expression levels and prognostic significance of GLS and GLS2 in ER+/HER2- patients in well-characterised DCIS and BC cohorts. 

## 2. Materials and Methods

### 2.1. Study Cohorts

Protein expression was conducted on two cohorts of ER+/HER2− BC comprising DCIS and IBC. Supplementary Tables S1 and S2 summarises the clinicopathological parameters of the two study cohorts. Patients were presented and managed at Nottingham City Hospital, Nottingham, UK. Clinicopathological, treatment and outcome data were collected and were prospectively maintained. 

The DCIS cohort included primary DCIS (*n* = 206), without synchronous IBC as previously described [17,18]. Clinicopathological data included age at diagnosis, method of diagnosis, either screening or symptomatic presentation, tumour grade, size and comedo-type necrosis. Expression of ER, progesterone receptor (PR), HER2 and Ki67 were previously determined for this cohort [18]. Local recurrence-free interval (LRFI) was defined as any event of ipsilateral local recurrence (either DCIS or IBC) occurring after 6 months from the primary treatment.

The IBC cohort includes a well-characterised series of tumours from patients (*n* = 717) with long-term follow-up [19]. Outcome data included recurrence-free interval (RFI) and BC specific survival (BCSS), which was defined as the time (in months) from the date of primary surgical treatment to the time of recurrence or death from BC, respectively.

### 2.2. Transcriptomic Data

*GLS* and *GLS2* gene copy number (CN) aberrations and gene expression were evaluated in a cohort of 1398 ER +/HER2- BC cases in the Molecular Taxonomy of Breast Cancer International Consortium (METABRIC) cohort. Data were pre-processed and normalised as described previously [20]. Dichotomisation of *GLS*/*GLS2* mRNA expression was determined using the mean value. In addition, Breast Cancer Gene-Expression Miner v4.5 (bc-GenExMiner v4.5) incorporating TCGA and SCAN-B RNA sequencing data (*n* = 4712) was used. Correlation between *GLS* and *GLS2* mRNA expression with glutamine-associated genes was also investigated. The selection of these genes was based on previous publications, as either regulatory genes or supporting the biological function of GLS or GLS2 in the glutamine metabolism [13,21,22,23].

### 2.3. Glutaminase Protein Expression

Prior to immunohistochemistry (IHC) staining, the specificity for rabbit monoclonal GLS (Clone EP7212, Abcam, UK) and GLS2 (Ab169954, Abcam Plc, Cambridge, UK) primary antibodies was validated by western blotting (WB) using IBC (MCF7, ZR751, BT474, MDA-MB-231, T47D, UACC-812, MDA-MB-175, BT549, HCC1500 and SKBR3) and DCIS (MCF10DCIS) lysates obtained from cells from the American Type Culture Collection (Rockville, MD, USA), as previously described [13]. GLS was used at a dilution of 1:1000 overnight at 4 °C, while GLS2 was diluted at 1:1500 and incubated for 1hr at room temperature. Mouse monoclonal anti-β-actin antibody (Sigma, Life Sciences) (1:5000) was included as a positive control. Donkey anti-rabbit and Donkey anti-mouse fluorescent secondary peroxidase-conjugated antibodies (1:15,000 IRDye 680RD and IRDye 800CW, LI-COR Biosciences) were applied for 1hr at room temperature. Images were detected using the LI-COR Odyssey Fc machine with Image Studio 4.0 (LI-COR Biosciences) at wavelengths 700 nm and 800 nm. Specific bands were observed at the predicted size of 73 kDa and 65 kDa corresponding to KGA and GAC isoforms of GLS and 65 kDa and 31 kDa corresponding to LGA and GAB isoforms of GLS2 (Supplementary Figure S1). In addition, peptide blocking using IHC was performed to validate the specificity of GLS and GLS2 antibodies (Supplementary Figure S2). GLS and GLS2 antibodies were incubated with GLS (Ab206976) and GLS2 (Ab169954) peptides, respectively (Abcam Plc, Cambridge UK). There were no visible bands in Western blotting and an absence of staining in IHC compared to the antibody alone, which displayed bands and positive staining. GLS2 antibody was additionally incubated with GLS peptide (1:2 ratio), which showed positive staining, further confirming that GLS and GLS2 antibodies do not cross-react. 

Tissue microarray (TMA) was constructed from both cohorts as previously described [17,24]. IHC was performed on 4μm TMA sections from both cohorts using the Novocastra Novolink TM Polymer Detection Systems Kit (Code: RE7280-K, Leica, Biosystems, UK) according to manufacturer instructions and as previously described [13]. Each antibody was used in a separate set of slides (non-dual staining). Heat-induced antigen epitope retrieval was performed in citrate buffer (pH 6.0) for 20min using a microwave oven (Whirlpool JT359 Jet Chef 1000 W) for both antibodies. Tissues were incubated with either GLS antibody 1:50 (Clone EP7212, Abcam, UK) or GLS2 antibody 1:400 (Ab169954, Abcam Plc, Cambridge, UK) diluted in Leica antibody diluent (RE AR9352, Leica Biosystems, Newcastle upon Tyne, UK) at 4 °C overnight and at room temperature for 1hr respectively. Negative (omission of the primary antibody) and positive control (liver tissue) were included according to the manufacturer’s datasheet.

### 2.4. Scoring of GLS and GLS2 Expression

Stained TMA slides were scanned using a high-resolution digital scanner (NanoZoomer; Hamamatsu Photonics, Welwyn Garden City, UK) at x20 magnification and viewed using Xplore viewing software (Philips Healthcare, Belfast, UK). Assessment of staining for GLS and GLS2 in DCIS and invasive BC was based on a semi-quantitative assessment using a modified histochemical score (H-score), which included an assessment of both the intensity of staining and the percentage of stained tumour cells. For the intensity, a score index of 0, 1, 2 and 3 which corresponded to negative, weak, moderate and strong staining, was used, and the percentage of positively stained tumour cells for each intensity was estimated subjectively. The final H-score was calculated by multiplying the percentage of positively stained cells (0–100) by the intensity (0–3), producing a total range of 0–300 [25]. A pathologist blind scored 10% of the cases for inter-observer concordance. GLS and GLS2 protein expression were dichotomised into a low and high expression using the median H-score as per previous publications [26,27]. Breast cancer luminal subtypes were defined based on the IHC profile as: luminal A: ER +/HER2- low proliferation (Ki67 < 10%) and luminal B: ER +/HER2- high proliferation (Ki67 > 10%). For GLS expression, an H-score of 20 for DCIS and 100 for IBC were used. The median H-score for GLS2 expression was 103 and 90 in DCIS and IBC, respectively. 

### 2.5. Statistical Analysis

SPSS version 25 (Chicago, IL, USA) was used to carry out statistical analyses. Continuous levels of *GLS* and *GLS2* mRNA and protein expressions were correlated with other parameters using Pearson’s correlation coefficient. Differences in the mean between three or more groups were assessed using one-way analysis of variance (ANOVA) with the post-hoc Tukey multiple comparison test (for normalised data), while Mann–Whitney and Kruskal–Wallis tests were applied for non-parametric data. Kaplan–Meier survival curves and a log-rank test were used to investigate the association of glutaminase mRNA/protein expression with the clinical outcome. The Cox regression model was applied for the multivariate analysis against LRFI. A two-tailed *p*-value < 0.05 for all the tests was considered significant. 

## 3. Results

### 3.1. Patterns of GLS and GLS2 Protein Expression

**Figure 1:**
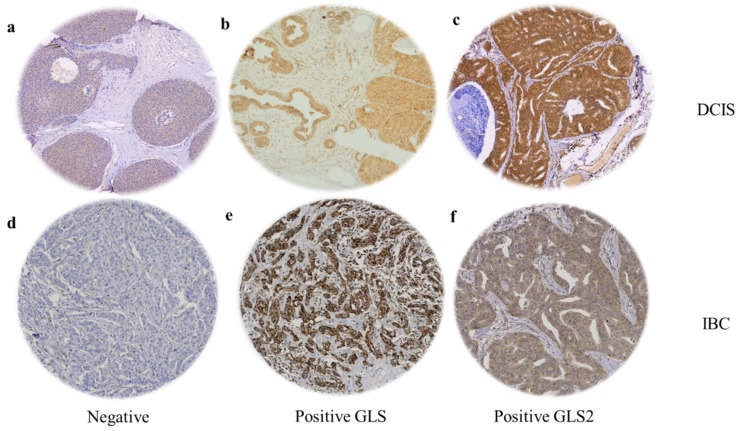
GLS and GLS2 protein expression in ER +/HER2- DCIS and invasive breast cancer. Representative TMA images (×20 magnification) depicting (**a**) negative immunostaining, positive GLS (**b**) and GLS2 (**c**) immunostaining in DCIS cases. (**d**) Negative immunostaining, positive GLS (**e**) and GLS2 (**f**) expression in invasive breast tumours.

When present, GLS and GLS2 were located predominantly in the cytoplasm of tumour cells of both DCIS and IBC, with intensity levels varying from low to high (Figure 1). GLS showed negative or faint staining in the adjacent apparently normal terminal duct lobular units (TDLUs), while GLS2 showed moderate expression. Occasional stained inflammatory cells and surrounding stromal fibroblasts were sometimes evident (Figure 1). GLS expression was significantly higher in IBC than DCIS (F = 332.4, *p* < 0.0001) and GLS2 was higher in DCIS than IBC (F = 9.8, *p* = 0.002).

### 3.2. Glutaminase Expression in ER +/HER2- DCIS 

**Figure 2:**
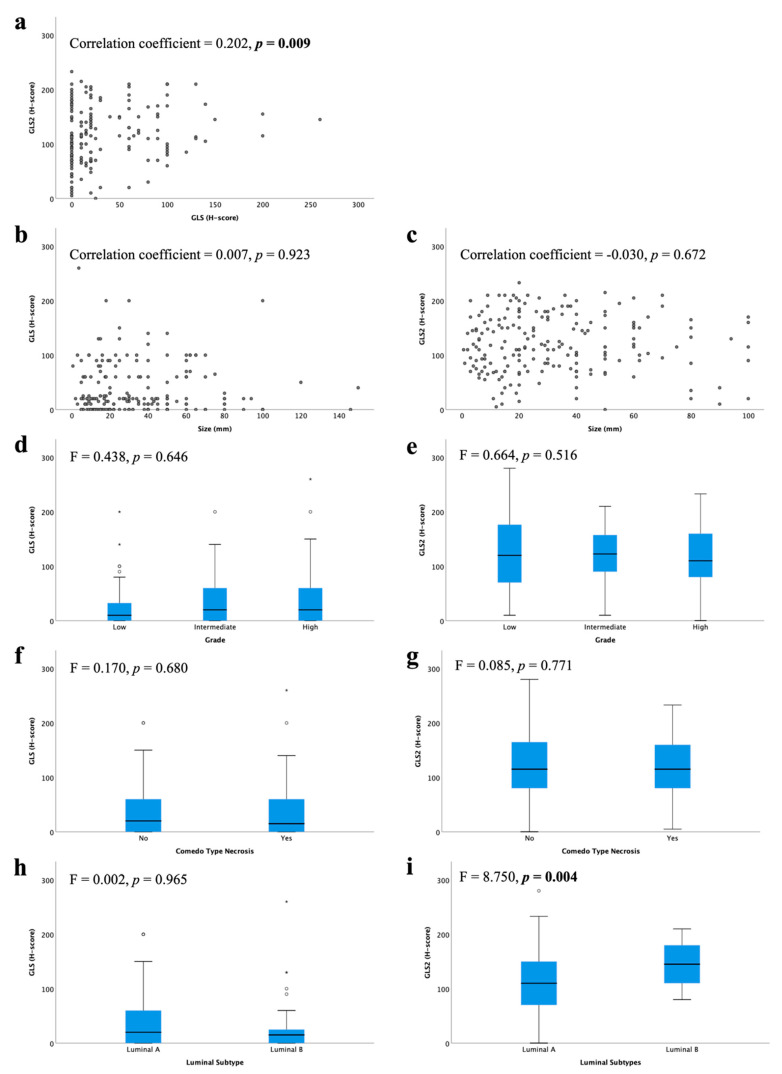
Glutaminase protein expression and its association with clinicopathological parameters and molecular subtypes in ER +/HER2- DCIS: (**a**) GLS and GLS2, GLS and (**b**) tumour size, (**d**) tumour grade, (**f**) comedo type necrosis, (**h**) luminal subtypes; GLS2 and (**c**) tumour size, (**e**) tumour grade, (**g**) comedo type necrosis, (**i**) luminal subtypes using Kruskal–Wallis test. Statistically significant *p* values are in **bold**.

There was a weak positive linear correlation between GLS and GLS2 protein expression (Figure 2a; *r* = 0.202, *p* = 0.009). However, there were no associations between GLS or GLS2 with other clinical parameters, including tumour size or DCIS grade (Figure 2b–g). There was no difference in GLS expression between luminal subtypes (Figure 2h), but GLS2 expression was significantly higher in luminal B compared with luminal A tumours (Figure 2i, *p* = 0.004). 

### 3.3. Glutaminase Expression in ER +/HER2- Invasive BC

**Figure 3:**
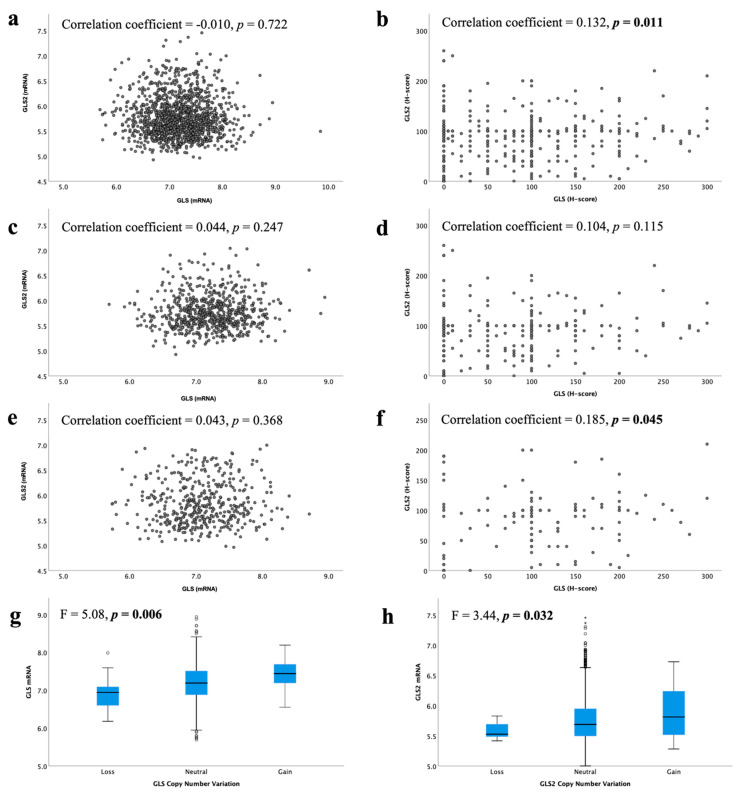
Correlation between glutaminase mRNA and protein expression in ER +/HER2- invasive breast cancer: *GLS* and *GLS2* mRNA in (**a**) all tumours, (**c**) luminal A tumours, (**e**) luminal B tumours; GLS and GLS2 protein in (**b**) all tumours, (**d**) low proliferation tumours, (**f**) high proliferation tumours. Copy number gain and relationship with mRNA expression for (**g**) *GLS* and (**h**) *GLS2* were analysed using Pearson’s correlation coefficient. Data represented with median ± standard deviation. Statistically significant *p* values are in **bold**.

In the METABRIC cohort, a total of 19/1398 IBC (1.4%) showed *GLS* CN gain, whereas 16 cases (1.1%) showed CN loss. Regarding *GLS2*, CN gain was observed in 50 cases (3.6%) and loss observed in only 7 cases (0.5%). There was an association between *GLS* and *GLS2* CN variations and their corresponding mRNA expression (Figure 3g, *p* = 0.006 and Figure 3h, *p* = 0.032, respectively).

At the mRNA level, there was no correlation between *GLS* and *GLS2* in ER +/HER2- BC, luminal A or luminal B tumours (Figure 3a,c,e, all *p* > 0.05). However, in the GeneMiner dataset, there was a very weak negative correlation between *GLS* and *GLS2* in all ER+ (*p* < 0.00001) and luminal A (*p* = 0.003) BC classes, but not the luminal B tumours (Supplementary Figure S3, *p* = 0.202). At the protein level, there was a weak positive linear correlation between GLS and the GLS2 protein in ER +/HER2- BC cases (Figure 3b, *p* = 0.011). When biological subtypes were considered, GLS and GLS2 proteins remained positively correlated, albeit weakly, in the high proliferation/luminal B tumours (Figure 3f, *p* = 0.045), but not the low proliferation/luminal A tumours (Figure 3d, *p* = 0.115). 

### 3.4. Association of Glutaminase with Clinicopathological Parameters in Invasive BC

**Figure 4:**
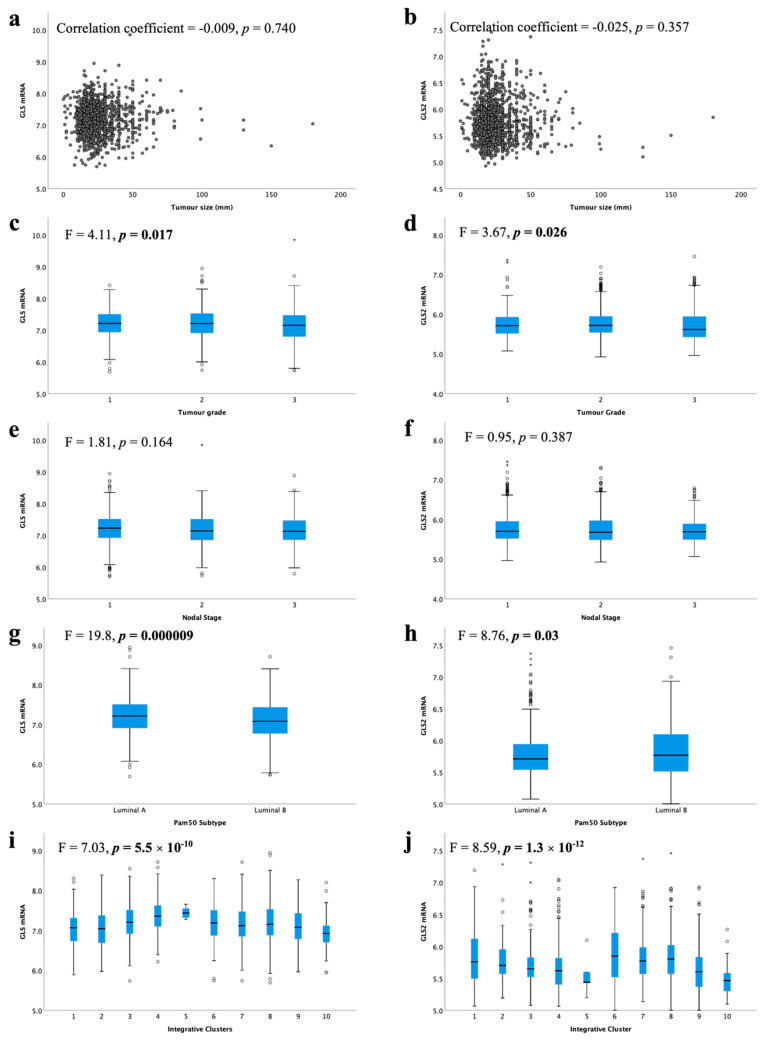
Glutaminase mRNA expression and its association with clinicopathological parameters: *GLS* and (**a**) tumour size, (**c**) tumour grade, (**e**) lymph node stage, (**g**) luminal subtypes, (**i**) METABRIC Integrative clusters; *GLS2* and (**b**) tumour size, (**d**) tumour grade, (**f**) lymph node stage, (**h**) luminal subtypes, (**j**) METABRIC Integrative clusters were analysed using Pearson’s correlation coefficient. Data represented with median ± standard deviation. Statistically significant *p* values are in **bold**.

High *GLS* and *GLS2* mRNA were associated with lower tumour grade (Figure 4c, *p* = 0.017, Figure 4d, *p* = 0.026, respectively). There was no association between *GLS* or *GLS2* mRNA expression with tumour size (Figure 4a,b) or nodal stage (Figure 4e,f). 

When comparing the levels of glutaminase mRNA expression in biological subtypes, there was a significantly lower level of *GLS* in luminal B compared with luminal A tumours (Figure 4g, *p* < 0.001). In contrast, luminal B tumours showed higher *GLS2* expression than luminal A tumours (Figure 4h, *p* = 0.03). Luminal B tumours were more likely to have *GLS* CNV, either gain or loss, compared with luminal A tumours (*p* = 0.019). Similarly, *GLS2* CN gains were primarily observed in luminal B tumours (*p* = 0.00004).

Within the METABRIC Integrative clusters, high *GLS* mRNA expression was associated with cluster 4 (predominately luminal A) (Figure 4i, *p* < 0.0001). In contrast, high *GLS2* was associated with cluster 6 (predominately luminal B) (Figure 4j, *p* < 0.0001). *GLS2* copy number gain was associated with cluster 1, which are predominantly luminal B tumours (*p* = 0.000006). There were no other associations between CN variations and Integrative clusters.

**Figure 5:**
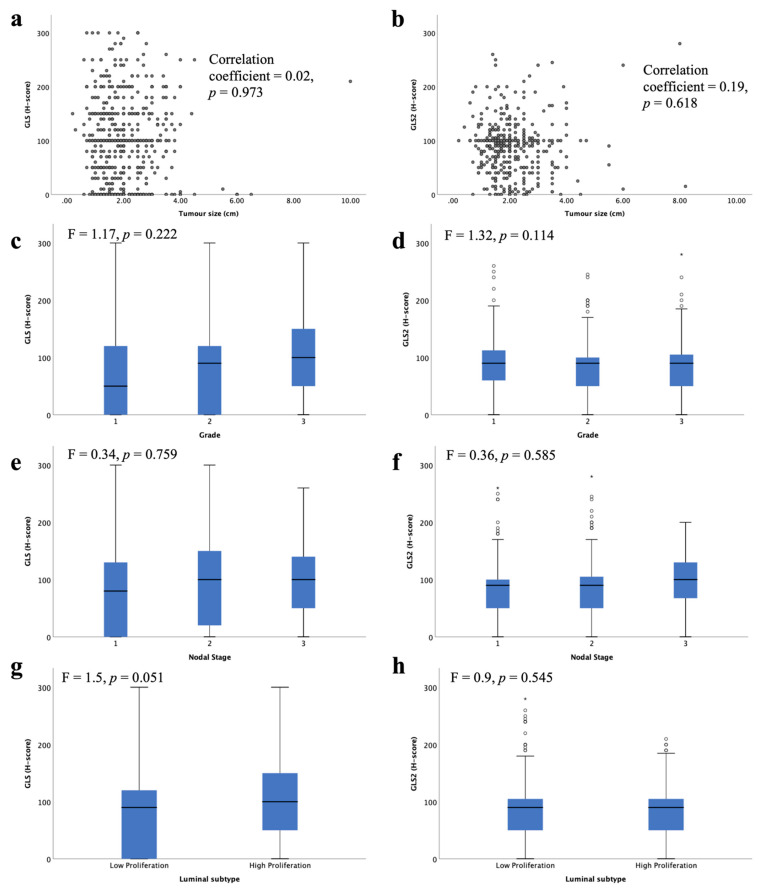
Glutaminase protein expression and its association with clinicopathological parameters and molecular subtypes in ER +/HER2- invasive breast cancer: GLS and (**a**) tumour size, (**c**), tumour grade, (**e**) lymph node stage, (**g**) luminal subtypes; GLS2 and (**b**) tumour size, (**d**) tumour grade, (**f**) lymph node stage, (**h**) luminal subtypes using one-way analysis of variance with the post-hoc Tukey test. Data represented with median ± standard deviation. Statistically significant *p* values are in **bold**.

GLS and GLS2 protein were not associated with any of the key clinicopathological parameters: tumour size, tumour grade or nodal stage (Figure 5a–f). There was a trend towards higher GLS protein expression in the high proliferative luminal tumours compared with the low proliferative tumours (Figure 5g, *p* = 0.051). There was no significant difference between GLS2 protein expression in the luminal subtypes (Figure 5b).

### 3.5. Glutaminase and Glutamine Metabolism-Related Genes and Proteins

There was a weak positive correlation between GLS and GLS2 with Glutamate Dehydrogenase (GLUD1) and the solute carriers (SLC38A2 and SLC7A8) at both the mRNA (Table 1 and Table 2, all *p* < 0.05) and protein levels (*p* < 0.01; Table 3 and Table 4). In addition, GLS mRNA and protein expression were weakly positively correlated with ALDH4A1 (Table 1 and Table 3, *p* < 0.001) and GLS2 was moderately correlated with the solute carrier (SLC7A11) at both mRNA and protein levels (Table 2 and Table 4, *p* < 0.01). 

GLS and GLS2 protein, but not mRNA, were also weakly positively correlated with c-MYC, SLC3A2 and enzymes involved in glutamine-proline regulatory axis (ALDH18A1 and PRODH) (Table 3 and Table 4, all *p* < 0.001). Additionally, there was a weak positive correlation between GLS and BRCA1 (*p* < 0.001), p53 (*p* < 0.01), PIK3CA (*p* < 0.001) and the key glutamine solute carriers (SLC1A5 and SLC7A5) (Table 3, all *p* < 0.001). GLS2 protein was weakly positively correlated with SLC3A2 (Table 4, *p* < 0.001).

With respect to the luminal subtypes, both luminal A (low proliferative) and B (high proliferative) tumours showed a weak positive correlation between GLS mRNA and protein expression with GLUD1 and SLC38A2 (Table 3 and Table 4, *p* < 0.01). GLS protein expression, but not mRNA, was also similarly weak to moderately positively correlated with ALDH18A1, ALDH4A1, c-MYC, PRODH, SLC3A2, SLC7A11 and SLC7A5 in both luminal subtypes (Table 3, all *p* ≤ 0.001). In addition, there was a weak positive correlation between GLS protein and BRCA1, p53, PIK3CA and SLC1A5 in low proliferative but not high proliferative luminal tumours (Table 3, all *p* ≤ 0.001). 

Both low and high proliferative luminal tumours showed weak positive correlation between GLS2 protein and ALDH18A1 (*p* < 0.001), ALDH4A1 (*p* < 0.01), ATF4 (*p* = 0.001), PRODH (*p* < 0.001), SLC38A2 (*p* ≤ 0.001) and SLC7A11 (Table 4, *p* < 0.001). Low proliferative, but not high proliferative, luminal tumours also showed a weak positive correlation between GLS2 protein and c-MYC (*p* < 0.001), SLC1A5 (*p* < 0.05) and SLC3A2 (Table 4, *p* < 0.001).

### 3.6. Glutaminase and Outcome in ER +/HER2- DCIS

**Figure 6:**
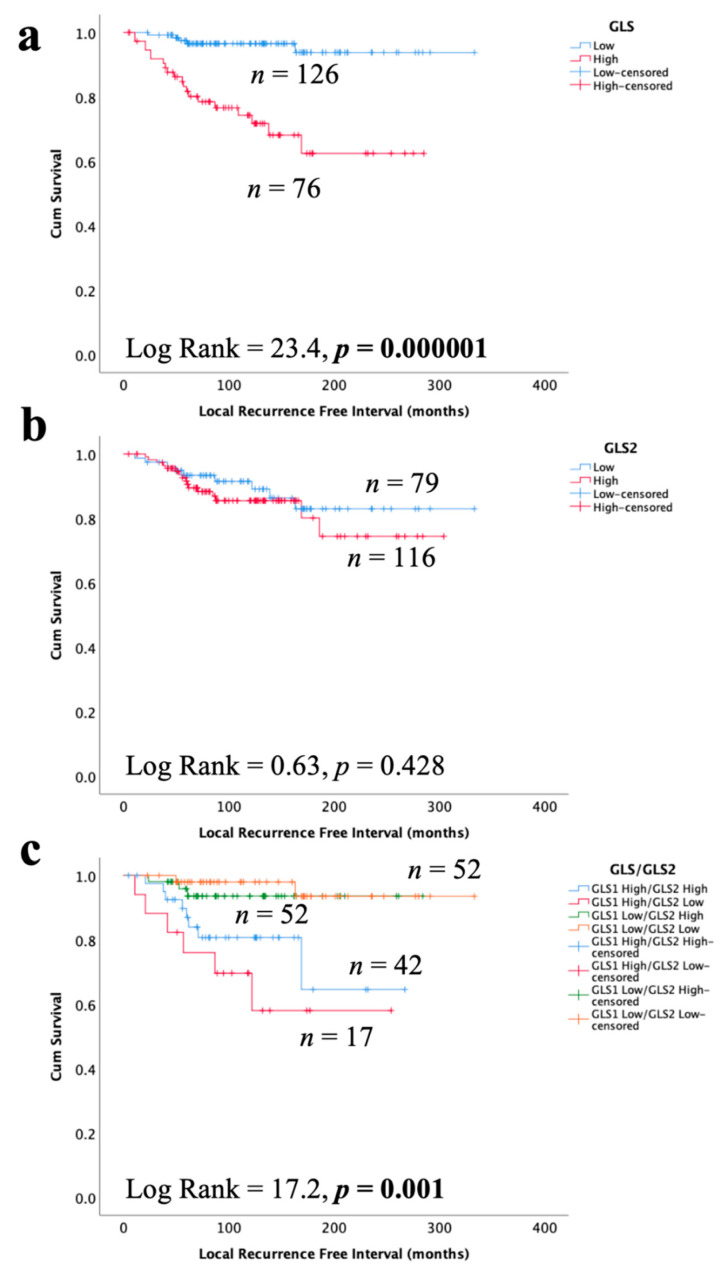
Kaplan–Meier of GLS and GLS2 protein expression with tumour recurrence in ER +/HER2- DCIS patients: (**a**) GLS, (**b**) GLS2, and (**c**) combined expression of GLS and GLS2. Statistically significant *p* values are in **bold**.

High GLS expression in DCIS was associated with shorter LRFI for all recurrences (Figure 6a, *p* < 0.0001), whereas there was no association between GLS2 and DCIS outcome (Figure 6b, *p* = 0.428). When stratifying patients, taking into account both GLS and GLS2 co-expression, DCIS with GLS high/GLS2 low expression was associated with the shortest LRFI with GLShigh/GLS2 high showing moderate outcome and those tumors without GLS expression, irrespective of GLS2 expression, having the best outcome (Figure 6c, *p* = 0.0001). In multivariate Cox regression, GLS and GLS/GLS2 co-expression remained predictors of shorter LRFI independent of tumour size, grade and comedo necrosis (Table 5, *p* = 0.0008 and *p* = 0.003, respectively).

### 3.7. Glutaminase and Outcome in ER +/HER2- Invasive BC

**Figure 7:**
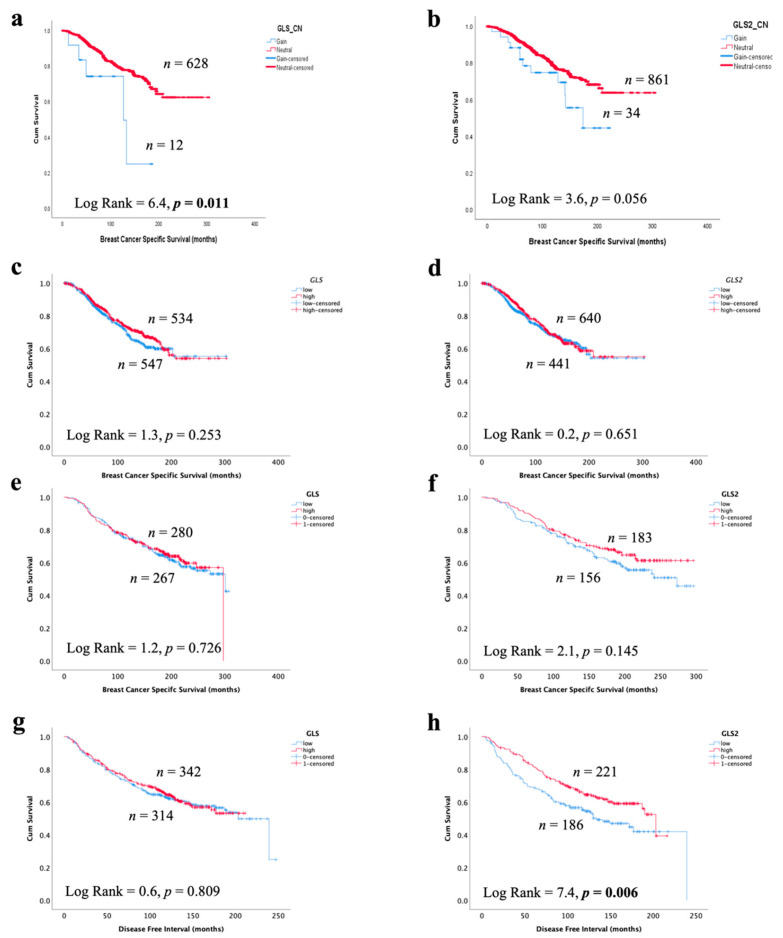
Association of glutaminase expression with patient outcome in ER +/HER2- invasive breast cancer in the METABRIC and Nottingham series: breast cancer-specific survival of (**a**) *GLS* copy number gain, (**b**) *GLS2* copy number gain, (**c**) *GLS* mRNA, (**d**) *GLS2* mRNA, (**e**) GLS protein and (**f**) GLS2 protein, a disease-free interval of (**g**) GLS protein and (**h**) GLS2 protein. Statistically significant *p* values are in **bold**.

In invasive breast cancer, CN gain of *GLS* and high *GLS* mRNA expression, but not *GLS2*, was associated with poor patient survival (Figure 7a,b, *p* = 0.011 and *p* = 0.056). There was no association between GLS or GLS2 mRNA or protein expression with patient BCSS (Figure 7c–f). Likewise, there was no association with *GLS* or *GLS2* mRNA with either patient survival or disease-free interval in GeneMiner (Supplementary Figure S4). However, GLS2 protein (*p* = 0.006), but not GLS, was predictive of a longer recurrence-free interval (Figure 7g,h), which remained independent of tumour size, grade and nodal stage (*p* = 0.003, Table 6).

## 4. Discussion

The glutamine metabolism is important in cancer cell proliferation and in promoting invasiveness [28]. It has been well established that glutamine synthesis is upregulated in most cancers, including BC, and consequently, glutaminase catalytic activity and levels are upregulated [5]. Several studies demonstrate that glutaminase contributes to cancer tumour growth in various human cancers such as prostate, lung and colorectal [7,8]. Despite these findings, the role of glutaminase in the progression of DCIS into the invasive disease stage remains poorly understood. In addition, studies on GLS2 expression in BC are limited. The current study evaluated the transcriptomic and proteomic expression of GLS and GLS2 and their association with various clinicopathological parameters and linked each biomarker to the patient outcome to provide an understanding of the prognostic significance of GLS and GLS2 in BC. To our knowledge, this is the first study to evaluate the role of both GLS and GLS2 in pre-invasive and invasive ER+/HER2- tumours. The ER +/luminal tumours are the most common type of BC, accounting for about 55–80% of all BC types and have varied tumour biology, disease prognosis and recurrence [29].

This study has revealed for the first time that that high GLS and GLShigh/GL2low expression are associated with shorter LRFI in DCIS independent from other clinicopathological variables. Our preliminary results, therefore, suggest that glutaminase Ftrcould be used as prognostic markers in early-stage disease to predict patient outcome, and this warrants further validation in external DCIS cohorts. The findings highlighted the importance of GLS in breast tumour proliferation and invasiveness and could potentially be used as a target for inhibition via the potent and non-competitive allosteric GLS inhibitor CB−839 (telaglenastat). CB-839 has anti-proliferative activity in triple-negative BC [7], and several clinical trials for solid cancers are ongoing [30]. In vitro and in vivo investigations using appropriate models are necessary to confirm this.

With respect to invasive tumours, increased expression of GLS2 protein predicted longer recurrence-free intervals. This finding concurs with previous results that GLS2 has been linked to a role in suppressing tumour growth. It has been demonstrated that overexpression of GLS2 decreases HCC cell invasiveness by counteracting the small GTPase Rac1 [31]. Nevertheless, consistent with a previous study, copy number gain of GLS was associated with poor outcomes in IBC [13].

Previous reports [5,7] have shown that high proliferative tumours such as TNBC and luminal B have higher glutamine metabolism and show increased activity of glutaminase compared to low proliferating tumours. It is noteworthy that although GLS and GLS2 catalyse the conversion of glutamine to glutamate, the expression and regulation of the two isozymes is distinct. The former is the frequently upregulated isoform in most cancers. This study showed a strong trend towards higher GLS protein expression in the high proliferative ER+ tumours in invasive breast cancer and in DCIS. In addition, High GLS2 protein expression was associated with luminal B compared to luminal A tumours in DCIS. When assessing the correlation between GLS and GLS2 and the two cohorts, we observed a positive correlation between the high expression of GLS and GLS2 protein in pre-invasive tumours and high proliferative invasive tumours. This finding could suggest that in both the pre-invasive stage and the invasive stage, tumour cells might be overcoming the effect of GLS2 overexpression by overexpressing GLS. However, further mechanistic studies for this scenario are highly warranted to understand the underlying molecular mechanisms.

The relationship between GLS and GLS2 and other regulatory genes at both mRNA and protein expression was also investigated. A weak positive correlation between glutaminase isozymes and c-Myc in luminal types at the protein level was observed. Evidence from various studies suggests that GLS is directly activated by c-Myc enabling sustained uncontrolled tumour cell proliferation. c-Myc is known as an important driver in maintaining a glutaminolysis phenotype, particularly in ER- tumours, and enhances GLS activity indirectly via suppressing the expression of miR-23a/b [21,32]. Our data suggest that this regulation might also occur in the ER +/luminal subtype. We observed a weak positive correlation between GLS with PI3KCa within the low proliferation subgroup. PI3KCa, a known oncogene, has a role in regulating cell proliferation and survival as well as an important role in regulating glucose and glutamine uptake and metabolism in different cancers. In BC, PIK3Ca mutations tend to be associated with hormone receptor-positive tumours, and a study carried out by Lau et al. has provided further evidence of the importance of PIK3Ca mutations in metabolic reprogramming, specifically increasing glutamine uptake and glutamate production by modulating pyruvate dehydrogenase activity [33]. *TP53* has been linked to regulating the glutamine metabolism by mediating the *GLS2* gene and having a tumour suppression effect on tumour cells [22]. Interestingly, in the current study, a weak positive association between wild type *TP53* and GLS expression in the low proliferating tumour was observed.

The association of both GLS and GLS2 with glutamine transporters and other enzymes involved in the glutamine metabolism is not surprising. Our analysis demonstrated weak to moderate associations between glutaminase with most of the glutamine metabolism-related enzymes and solute carriers. Among these is GLUD1, which was weakly associated with GLS and GLS2 at both mRNA and protein levels in the low and high proliferative tumours. Craze and colleagues [34] have shown that there is a relationship between GLUD1 and luminal tumours compared to HER2+ tumours. Furthermore, a weak positive correlation with both ALDH18A1 and PRODH was observed. Previously Craze et al. demonstrated that these enzymes were highly expressed in a subset of ER+ tumours that have high proliferation and were related to poor patient outcomes [13]. We also show that high expression of GLS and GLS2 were weakly associated with high expression of SLC7A5, SLC3A2 and SLC1A5. In their findings, El Ansari et al. demonstrated that the combination of SLC1A5, SLC7A5 and SLC3A2, defined as high SLCs cluster, was associated with poor prognostic markers in highly proliferative ER-positive tumours [23]. Our observation in this subset of BC is consistent with the previous studies. Although our findings did not show correlation with key clinicopathological features in invasive BC, mostly association with glutamine-metabolism-related genes, the findings may suggest that glutaminase isozymes expression in this subset of breast cancer is important in tumour biology rather than a clinical outcome in invasive BC. Further investigation studies are needed to understand the underlying molecular mechanisms.

## 5. Conclusions

This study provides strong evidence for the use of GLS as a prognostic biomarker for invasive progression of luminal DCIS and a potential target for inhibition. Further validation in external cohorts is warranted along with functional studies to decipher the role of GLS and its mechanism of action as a driver of disease progression.

## Figures and Tables

**Table 1:** Correlation of *GLS* mRNA expression with glutamine-metabolism-related mRNA in invasive luminal breast cancer.

*GLS vs*	ER+/HER2- (*n* = 1398)		Luminal A (*n* = 693)		Luminal B (*n* = 443)	
	Correlation Coefficient	*p* Value	Correlation Coefficient	*p* Value	Correlation Coefficient	*p* Value
*AKT1*	−0.012	0.675	−0.034	0.376	−0.005	0.923
*ALDH18A1*	0.040	0.139	0.066	0.081	0.026	0.591
*ALDH4A1*	0.101	**0.000148**	0.136	**0.000331**	0.006	0.908
*ATF4*	0.031	0.245	0.042	0.272	0.095	**0.046**
*BRCA1*	−0.054	0.044	0.064	0.092	0.022	0.642
*c-MYC*	0.018	0.497	−0.005	0.903	0.070	0.141
*GLUD1*	0.141	**1.24 × 10^−7^**	0.147	**0.0001**	0.168	**0.0004**
*GLUL*	−0.138	**2.25 × 10^−7^**	−0.194	**2.84 × 10^−7^**	−0.109	0.022
*MTOR*	0.028	0.292	0.044	0.244	−0.047	0.324
*p53*	−0.169	**<0.0001**				
*PIK3CA*	0.047	0.078	0.117	**0.002**	0.058	0.220
*PRODH*	−0.210	0.425	−0.052	0.175	−0.092	0.054
*PYCR1*	−0.097	**0.000282**	−0.010	0.799	−0.124	**0.009**
*SLC1A1*	−0.025	0.349	−0.007	0.857	−0.048	0.316
*SLC1A2*	0.022	0.421	0.071	0.063	0.064	0.179
*SLC1A3*	0.066	**0.014**	0.094	**0.013**	0.060	0.209
*SLC1A5*	−0.135	**3.78 × 10^−7^**	−0.078	**0.040**	−0.183	**0.0001**
*SLC1A6*	−0.24	0.363	0.010	0.803	0.043	0.370
*SLC1A7*	0.010	0.713	0.038	0.319	−0.092	0.054
*SLC38A1*	0.049	0.066	0.057	0.131	0.052	0.272
*SLC38A2*	0.191	**6.33 × 10^−13^**	0.250	**2.24 × 10^−11^**	0.127	**0.007**
*SLC38A3*	0.041	0.124	0.141	**0.0002**	−0.047	0.326
*SLC38A5*	−0.004	0.893	0.056	0.140	−0.031	0.511
*SLC38A7*	0.157	**3.95 × 10^−9^**	0.219	**5.69 × 10^−9^**	0.169	**0.0004**
*SLC38A8*	−0.052	0.053	−0.049	0.201	0.009	0.849
*SLC3A2*	−0.144	**6.06 × 10^−8^**	−0.148	**0.00009**	−0.052	0.276
*SLC6A19*	−0.056	0.035	−0.018	0.641	−0.050	0.296
*SLC7A11*	0.014	0.604	−0.013	0.735	0.068	0.151
*SLC7A5*	−0.230	0.383	0.032	0.407	0.052	0.278
*SLC7A6*	0.180	**1.32 × 10^−11^**	0.220	**4.55 × 10^−9^**	0.107	0.024
*SLC7A7*	−0.99	**0.000218**	−0.109	**0.004**	−0.075	0.113
*SLC7A8*	0.065	**0.016**	0.063	0.096	0.075	0.113
*SLC7A9*	−0.005	0.862	−0.021	0.588	−0.007	0.888

Statistically significant *p* values are in **bold**.

**Table 2:** Correlation of *GLS2* mRNA expression with glutamine-metabolism-related mRNA in invasive luminal breast cancer.

*GLS2 vs*	ER +/HER2- (*n* = 1398)		Luminal A (*n* = 693)		Luminal B (*n* = 443)	
	Correlation Coefficient	*p* Value	Correlation Coefficient	*p* Value	Correlation Coefficient	*p* Value
***AKT1***	0.038	0.157	0.066	0.083	0.021	0.656
***ALDH18A1***	−0.001	0.958	0.030	0.427	−0.035	0.463
***ALDH4A1***	−0.055	**0.042**	−0.037	0.325	−0.037	0.435
***ATF4***	0.042	0.120	0.072	0.058	−0.001	0.987
***BRCA1***	0.203	**1.72 × 10^−14^**	0.120	**0.002**	0.169	**0.0003**
***c-MYC***	−0.104	**0.0001**	−0.150	**0.00007**	−0.040	0.399
***GLUD1***	0.224	**2.55 × 10^−17^**	0.242	**1.02 × 10^−10^**	0.151	**0.001**
***GLUL***	−0.017	0.519	−0.047	0.219	−0.055	0.246
***MTOR***	0.000	1.000	0.053	0.167	−0.052	0.279
***p53***	0.034	0.200				
***PIK3CA***	−0.12	0.649	0.016	0.674	−0.090	0.059
***PRODH***	-0.148	**2.69 × 10^−8^**	-0.114	**0.003**	-0.114	**0.016**
***PYCR1***	0.009	0.731	−0.016	0.672	−0.031	0.516
***SLC1A1***	0.070	**0.009**	0.046	0.224	0.035	0.468
***SLC1A2***	0.191	**6.14 × 10^−13^**	0.205	**5.28 × 10^−8^**	0.146	**0.002**
***SLC1A3***	−0.190	**8.55 × 10^−13^**	−0.163	**0.00002**	−0.249	**1.11 × 10^−7^**
***SLC1A5***	0.211	**1.42 × 10^−15^**	0.249	**2.87 × 10^−11^**	0.157	**0.001**
***SLC1A6***	−0.012	0.655	0.005	0.898	−0.007	0.889
***SLC1A7***	−0.041	0.127	−0.060	0.112	−0.035	0.461
***SLC38A1***	−0.075	**0.005**	−0.110	**0.004**	−0.132	**0.005**
***SLC38A2***	−0.021	0.425	−0.017	0.654	−0.014	0.766
***SLC38A3***	0.095	**0.0004**	0.125	**0.001**	0.077	0.104
***SLC38A5***	−0.102	**0.0001**	−0.105	**0.006**	−0.098	**0.038**
***SLC38A7***	0.029	0.281	0.079	0.037	0.058	0.226
***SLC38A8***	0.051	0.055	0.036	0.348	0.024	0.615
***SLC3A2***	0.018	0.495	−0.039	0.308	0.014	0.769
***SLC6A19***	0.002	0.930	−0.032	0.406	−0.007	0.880
***SLC7A11***	0.089	0.001	0.091	0.017	0.085	0.075
***SLC7A5***	−0.038	0.158	−0.008	0.826	−0.062	0.190
***SLC7A6***	−0.103	0.158	−0.054	0.158	−0.031	0.522
***SLC7A7***	−0.200	**4.14 × 10^−14^**	−0.211	**1.97 × 10^−8^**	−0.257	**4.29 × 10^−8^**
***SLC7A8***	0.061	**0.022**	0.074	0.500	0.020	0.681
***SLC7A9***	0.008	0.761	0.032	0.406	−0.095	0.045

Statistically significant *p* values are in **bold**.

**Table 3:** Correlation of GLS protein expression with glutamine-metabolism-related proteins in invasive luminal breast cancer.

GLS vs	ER +/HER2- (*n* = 668)		Low Proliferation (*n* = 275)		High Proliferation (*n* = 124)	
	Correlation Coefficient	*p* Value	Correlation Coefficient	*p* Value	Correlation Coefficient	*p* Value
pAKTs473	−0.003	0.958	0.006	0.913	0.017	0.842
ALDH18A1	0.347	**9.77 × 10^−17^**	0.303	**1.02 × 10^−8^**	0.348	**0.000002**
ALDH4A1	0.235	**4.53 × 10^−8^**	0.249	**0.000004**	0.232	**0.002**
ATF4	0.224	**1.34 × 10^−7^**	0.205	**0.0001**	0.244	**0.001**
BRCA1	0.151	**0.0005**	0.186	**0.001**	0.115	0.129
c-MYC	0.256	**1.42 × 10^−9^**	0.240	**0.000005**	0.288	**0.0001**
GLUD1	0.170	**0.00002**	0.142	**0.004**	0.230	**0.001**
MTORC1	−0.032	0.463	0.009	0.864	−0.038	0.622
p53	0.137	**0.007**	0.227	**0.0004**	0	1
PIK3CA	0.231	**0.000002**	0.239	**0.00009**	0.121	0.152
PRODH	0.312	**2.60 × 10^−12^**	0.308	**4.04 × 10^−8^**	0.323	**0.00003**
PYCR1	0.023	0.652	0.076	0.222	−0.086	0.340
SLC1A5	0.244	**1.57 × 10^−9^**	0.250	**6.96 × 10^−7^**	0.141	0.050
SLC38A2	0.293	**1.08 × 10^−8^**	0.188	**0.004**	0.333	**0.0003**
SLC3A2	0.355	**2.56 × 10^−16^**	0.387	**5.96 × 10^−13^**	0.269	**0.001**
SLC7A11	0.395	**1.34 × 10^−16^**	0.402	**8.22 × 10^−12^**	0.503	**1.78 × 10^−10^**
SLC7A5	0.236	**3.87 × 10^−7^**	0.188	**0.001**	0.256	**0.002**
SLC7A8	0.201	**0.001**	0.153	0.051	0.119	0.302

Statistically significant *p* values are in **bold**.

**Table 4:** Correlation of GLS2 protein expression with glutamine-metabolism-related proteins in invasive luminal breast cancer.

GLS2 vs	ER +/HER2- (*n* = 415)		Low Proliferation (*n* = 275)		High Proliferation (*n* = 124)	
	Correlation Coefficient	*p* Value	Correlation Coefficient	*p* Value	Correlation Coefficient	*p* Value
pAKTs473	−0.061	0.295	−0.149	**0.038**	0.084	0.441
ALDH18A1	0.342	**6.16 × 10^−12^**	0.263	**0.00002**	0.050	**8.35 × 10^−9^**
ALDH4A1	0.229	**0.000007**	0.210	**0.001**	0.292	**0.002**
ATF4	0.261	**8.71 × 10^−7^**	0.227	**0.001**	0.323	**0.001**
BRCA1	0.049	0.372	0.040	0.551	0.055	0.584
c-MYC	0.205	**0.0001**	0.230	**0.0004**	0.184	0.060
GLUD1	0.176	**0.0005**	0.106	0.089	0.311	**0.001**
MTORC1	0.069	0.204	0.029	0.655	0.142	0.171
p53	−0.042	0.508	0.023	0.766	0.039	0.742
PIK3CA	0.071	0.234	0.0125	0.091	0.059	0.586
PRODH	0.358	**4.18 × 10^−11^**	0.353	**1.76 × 10^−7^**	0.378	**0.0001**
PYCR1	0.082	0.196	0.067	0.383	0.117	0.317
SLC1A5	0.061	0.232	0.128	**0.041**	−0.024	0.799
SLC38A2	0.284	**1.04 × 10^−7^**	0.234	**0.001**	0.376	**0.00007**
SLC3A2	0.178	**0.0004**	0.273	**0.000009**	0.106	0.253
SLC7A11	0.285	**7.19 × 10^−8^**	0.266	**0.00005**	0.339	**0.0004**
SLC7A5	0.029	0.579	0.021	0.747	0.075	0.433
SLC7A8	0.175	**0.006**	0.156	0.051	0.229	0.048

Statistically significant *p* values are in **bold**.

**Table 5:** Multivariate survival analysis of variables predicting LRFI for GLS and GLS/GLS2 protein co-expression in ER +/HER2- DCIS.

Parameters	Hazard Ratio (HR)	95% Confidence Interval (CI)		*p* Value
		Lower	Upper	
GLS	7.4	2.7	20.0	**0.0008**
Comedo type necrosis	0.7	0.3	1.6	0.397
Tumour size	0.5	0.2	1.1	0.080
Tumour grade	1.3	0.6	2.4	0.445
GLS/GLS2 co-expression	0.5	0.4	0.8	**0.003**
Comedo type necrosis	0.9	0.3	2.7	0.884
Tumour size	0.5	0.2	1.2	0.111
Tumour grade	1.1	0.5	2.4	0.745

Statistically significant *p* values are in **bold**. DCIS, ductal carcinoma in situ.

**Table 6:** Multivariate survival analysis of variables predicting DFI for GLS2 protein expression in ER +/HER2- invasive breast cancer.

Parameters	Hazard Ratio (HR)	95% Confidence Interval (CI)		*p* Value
		Lower	Upper	
GLS2	0.6	0.5	0.9	**0.003**
Tumour size	1.3	0.9	1.7	0.141
Tumour grade	1.3	1.0	1.6	**0.033**
Nodal stage	1.5	1,2	1.9	**0.0003**

Statistically significant *p* values are in **bold**.

## Data Availability

The authors confirm that the datasets used and analysed during the current study are available from the corresponding author on reasonable request.

## Supplementary Materials

The following are available online at https://www.mdpi.com/article/10.3390/cancers13163963/s1, Figure S1: Western Blotting validation of GLS and GLS2 antibodies in breast cancer cell lines; Figure S2: Representative images of GLS and GLS2 Western blot and IHC peptide blocking; Figure S3: Correlation between GLS and GLS2 mRNA expression in ER+ invasive breast cancer using GeneMiner, Figure S4: Glutaminase mRNA expression and patient survival; Table S1: Clinicopathological parameters of ER+/HER2- of the breast cancer METABRIC and Nottingham series; Table S2: Summary of the clinicopathological characteristics of ER+HER2- DCIS patient cohort.
