# Supplementary material for: The Biological and Clinical Significance of Glutaminase in Luminal Breast Cancer
Source: Cancers (Basel). 2021 Aug 6;13(16):3963. doi: 10.3390/cancers13163963 (PMC8391318; doi:10.3390/cancers13163963)
Supplement: Supplementary file 1 [file cancers-13-03963-s001.zip › cancers-1273931-supplementary.pdf]

cancers

MDPI

The Biological and Clinical Significance of Glutaminase in Luminal Breast Cancer

Brendah K. Masisi, Rokaya El Ansari, Lutfi Alfarsi, Madeleine L. Craze, Natasha Jewa, Andrew Oldfield, Hayley Cheung, Michael Toss, Emad A. Rakha and Andrew R. Green

Table S1. Clinicopathological parameters of ER+/HER2- of the breast cancer METABRIC and Nottingham series.

	METABRIC (*n* = 1398)	Nottingham (*n* = 717)
Age		
<50	199 (14.5)	212 (29.6)
≥50	1174 (85.5)	505 (70.4)
Grade		
1	163 (12.2)	137 (19.1)
2	674 (50.6)	324 (45.3)
3	495 (37.2)	255 (35.6)
Size		
<2cm	449 (32.5)	378 (52.7)
≥2cm	932 (67.5)	339 (47.3)
Nodal Stage		
1	771 (55.2)	436 (60.9)
2	428 (30.7)	229 (32.0)
3	197 (14.1)	51 (7.1)
Histological type		
Ductal	1155 (82.6)	591 (82.4)
Lobular	206 (14.7)	93 (13.0)
Medullary	5 (0.4)	3 (0.4)
Miscellaneous	23 (1.6)	1 (0.1)
Special type	3 (0.2)	29 (4.0)
Progesterone Receptor		
Negative	425 (30.4)	144 (20.5)
Positive	973 (69.6)	559 (79.5)
PAM50		
Basal	37 (2.7)	
HER2	65 (4.7)	
Luminal A	693 (49.8)	N/A
Luminal B	443 (31.7)	
Normal	154 (11.1)	
IHC subtype		
Low Proliferation	N/A	221 (31.9)
High Proliferation		472 (68.1)

Table S2. Summary of the clinicopathological characteristics of ER+HER2- DCIS patient cohort.

Clinicopathological Parameters	GLS Expression	GLS2 Expression
	**Total (*n* = 206)**	**Total (*n* = 193)**
Age		
≤50	67(32.5)	61(31.6)
>50	139(67.5)	132(68.4)
DCIS Presentation		
Screening	79(38.3)	75(38.9)
Symptomatic	127(61.7)	118(61.1)
Comedo necrosis		
No	85(41.3)	84(43.5)
Yes	121(58.7)	109(56.5)
DCIS size		
≤2 cm	88(42.7)	79(40.9)
> 2 cm	118(57.3)	114(59.1)
Nuclear grade		
1	34(16.5)	33(17.1)
2	62(30.1)	61(31.6)
3	110(53.4)	99(51.3)

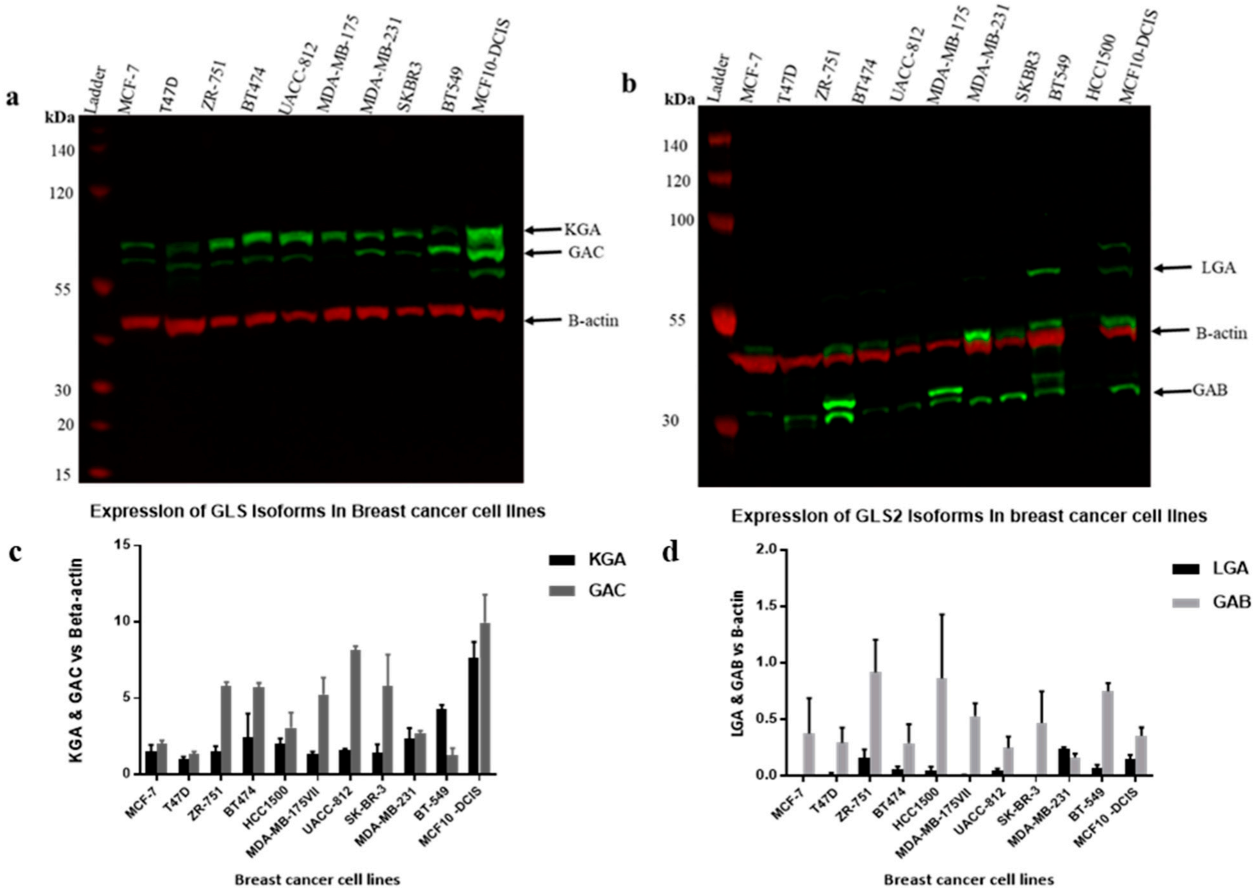

Figure S1. Western Blotting validation of GLS and GLS2 antibodies in breast cancer cell lines. Antibody validation of (**a**) specific bands visualised at the predicted molecular weight of (72KDa and 65KDa) representing the isoforms, KGA and GAC, of GLS respectively. (**b**) Specific bands were observed at the correct molecular weights for GLS2 (65kDa, 31kDa) and additional 35kDa band was observed which represents an alternate isoform of the protein. An additional band was also observed at approximately 45kDa which is not representative of the other known isoforms of GLS2 (present at 20kDa and 37kDa). Bar graph (**c** and **d**) shows ratio of expression of GLS and GLS2 isoforms and Beta-actin respectively. Results shown are mean +/- SE of at least two independent experiments performed in triplicates.

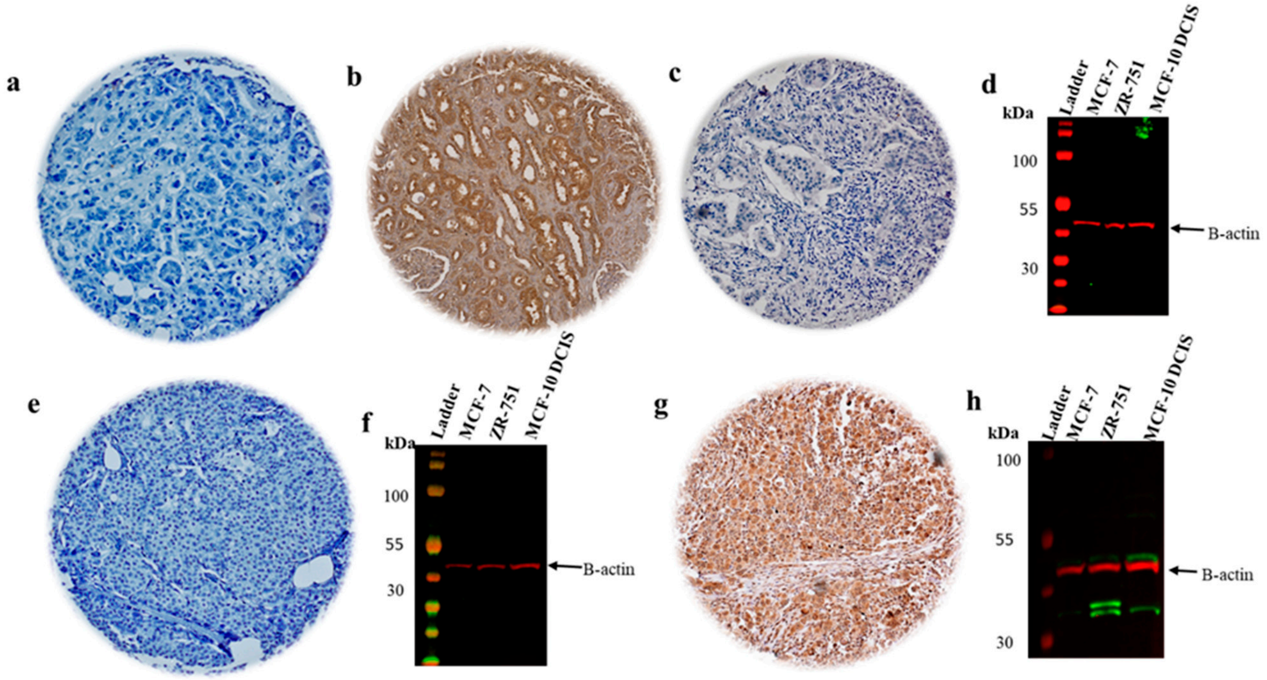

Figure S2. Representative images of GLS and GLS2 Western blot and IHC peptide blocking. (**a**) Negative (omission of the primary antibody) (**b**) positive control. Cores were stained with GLS antibody and GLS peptide (**c**), GLS2 antibody and GLS2 peptide (**e**) and GLS2 antibody and GLS peptide (**g**). (**d**, **f**, **h**) are the corresponding western blots.

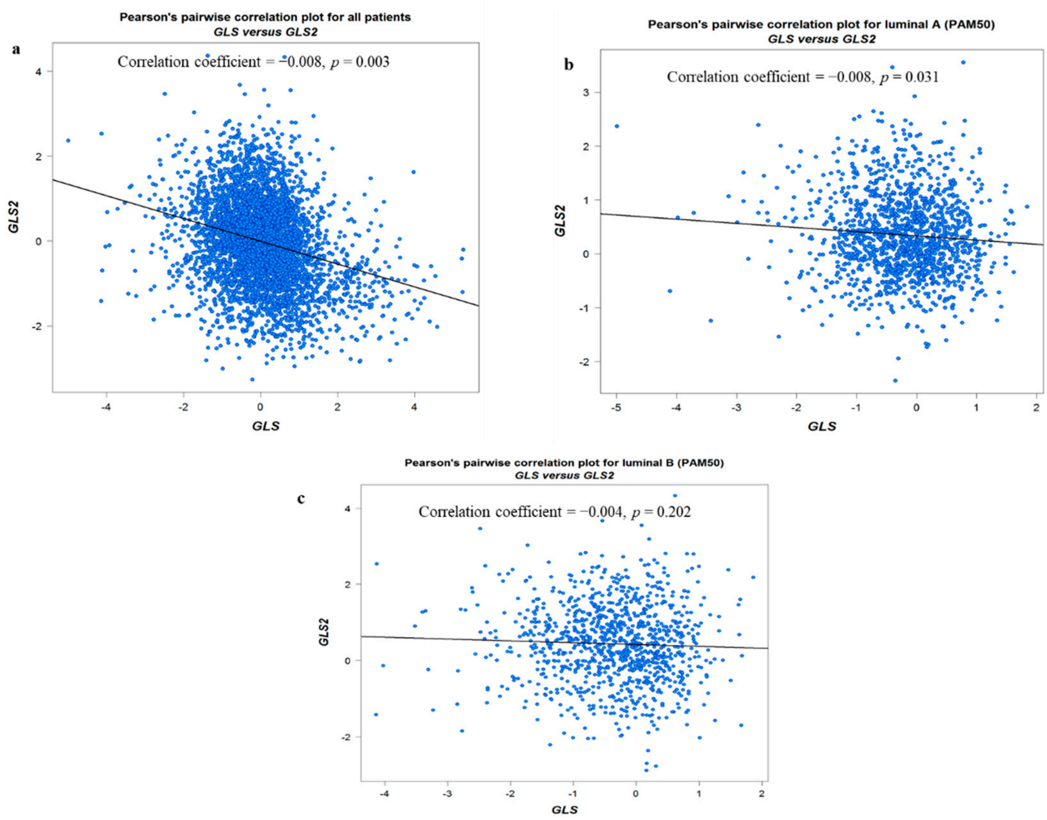

Figure S3. Correlation between *GLS* and *GLS2* mRNA expression in ER+ invasive breast cancer using GeneMiner (TCGA/SCAN-B) (**a**) all cases, (**b**) luminal A tumours, (**c**) luminal B tumours.

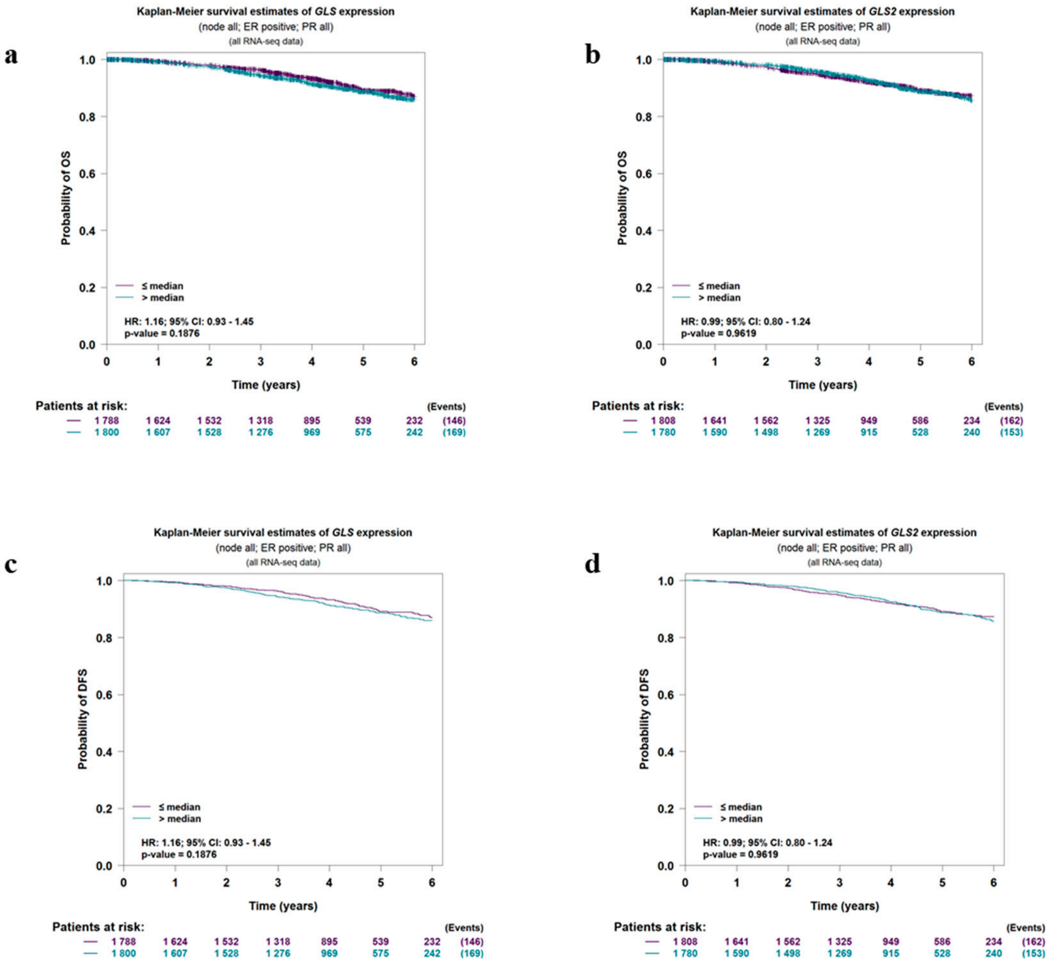

Figure S4. Glutaminase mRNA expression and patient survival (**a**) *GLS*, (**b**) *GLS2* and disease-free survival (**c**) *GLS*, (**d**) *GLS2* in ER+ invasive breast cancer using GeneMiner (TCGA/SCAN-B).
